# Heart Rate Variability and Cardiovascular Reflex Tests for Assessment of Autonomic Functions in Preeclampsia

**DOI:** 10.1155/2018/8163824

**Published:** 2018-09-18

**Authors:** Meenakshi Chaswal, Raj Kapoor, Achla Batra, Savita Verma, Bhupendra S. Yadav

**Affiliations:** ^1^Department of Physiology, VMMC and Safdarjung Hospital, New Delhi, India; ^2^Department of Obstetrics and Gynaecology, VMMC and Safdarjung Hospital, New Delhi, India; ^3^Department of Obstetrics and Gynaecology, SHMMH Medical college, Saharanpur, UP, India; ^4^Department of Physiology, BHU, Uttar Pradesh, India

## Abstract

Alterations in the autonomic cardiovascular control have been implicated to play an important etiologic role in preeclampsia. The present study was designed to evaluate autonomic functions in preeclamptic pregnant women and compare the values with normotensive pregnant and healthy nonpregnant controls. Assessment of autonomic functions was done by cardiovascular reflex tests and by analysis of heart rate variability (HRV). Cardiovascular reflex tests included deep breathing test (DBT) and lying to standing test (LST). HRV was analyzed in both time and frequency domain for quantifying the tone of autonomic nervous system to the heart. The time domain measures included standard deviation of normal R-R intervals (SDNN) and square root of mean squared differences of successive R-R intervals (RMSSD). In the frequency domain we measured total power (TP), high frequency (HF) power, low frequency (LF) power, and LF/HF ratio. Cardiovascular reflex tests showed a significant parasympathetic deficit in preeclamptic women. Among parameters of HRV, preeclamptic group had lower values of SDNN, RMSSD, TP, HF, and LF (ms^2^) and higher value of LF in normalised units along with high LF/HF ratio compared to normotensive pregnant and nonpregnant controls. Furthermore, normotensive pregnant women had lower values of SDNN, TP, and LF component in both absolute power and normalised units compared to nonpregnant females. The results confirm that normal pregnancy is associated with autonomic disturbances which get exaggerated in the state of preeclampsia.

## 1. Introduction

Preeclampsia, a syndrome affecting 5% to 7% of pregnancies, is characterised by new onset hypertension and proteinuria that develop after 20 weeks of gestation in a previously normotensive and nonproteinuric woman [1]. It is a significant cause of maternal and fetal morbidity and mortality [2]. Unfortunately, the precise pathophysiology of this multifaceted disorder remains elusive. Previous studies have demonstrated that an altered autonomic function may play a pivotal role in the development of preeclampsia. Although different methods have been used to assess autonomic cardiovascular control in preeclampsia, data available is scant and provides conflicting information about the status of autonomic functions in preeclamptic pregnancy [3–6]. Some studies have shown evidence for a role of autonomic dysfunction in preeclampsia [3–5], but the study by Eneroth and Storck [6] demonstrated no significant difference between preeclamptic women and normotensive pregnant women regarding the frequency domain parameters of HRV. Furthermore, the role of two divisions of autonomic nervous system in preeclampsia remains controversial with one study finding an increase in sympathetic activity in preeclampsia [7], whereas another investigation described it as a state of sympathetic hyperactivity with decreased parasympathetic control of heart rate [8]. Assessment of cardiac autonomic functions can be done by combination of cardiovascular reflex tests and heart rate variability (HRV). Both of these tests are quantitative, reproducible, noninvasive, and safe with minimal risk, if any, for the mother and the fetus [9]. HRV is a measure of cardiac autonomic tone and cardiovascular reflex tests measure reflex changes in heart rate and blood pressure in response to standardised stimuli and thus assess cardiovascular reactivity to stress. Combination of HRV and cardiovascular reflex tests can provide an extensive, comprehensive assessment of cardiac autonomic functions. However, to the best of our knowledge there has been no previous study using combination of HRV and autonomic cardiovascular reflex tests for assessment of autonomic functions in preeclampsia. The present investigation was thus designed to elucidate the extent and pattern of autonomic dysfunction in preeclampsia using both HRV and conventional cardiovascular reflex tests and to compare these cardiovascular indices with those in normal pregnant and nonpregnant females. In cardiovascular reflex tests, we evaluated cardiovagal function by measuring heart rate response to deep breathing and standing, whereas blood pressure response to standing was measured as an index of adrenergic function [9]. The results of the present study may lead to better understanding of the cardiovascular regulation in preeclampsia and may possibly determine whether autonomic tests could be useful to distinguish between normal and hypertensive pregnancy and whether they could be of relevance in the early identification of patients with an increased risk for preeclampsia so that best prenatal care for both the mother and her baby can be provided.

## 2. Methods

### 2.1. Subjects

The study participants included 120 women: 40 women with preeclampsia, 40 normotensive pregnant women who matched preeclamptic women with respect to age, period of gestation, and body mass index, and 40 healthy nonpregnant women of similar age. Preeclampsia was defined as the occurrence of hypertension (systolic blood pressure ≥140 mm Hg or diastolic blood pressure ≥ 90 mm Hg) after 20 weeks of gestation in a woman who is normotensive before, and proteinuria (presence of 300 mg or more of protein in 24 h urine sample or ≥ 2+ on dipstick) [10]. The exclusion criteria were multiple pregnancy, diabetes mellitus, chronic hypertension, liver disease, thyroid disorder, autoimmune disease, renal disease, and inflammatory conditions. All pregnant females were recruited from antenatal clinic of Department of Obstetrics and Gynaecology, VMMC & Safdarjung Hospital and healthy nonpregnant controls were randomly selected from among staff of the hospital. The cardiac autonomic function tests were carried in the Department of Physiology, VMMC & Safdarjung Hospital, New Delhi, India.

### 2.2. Study Design

The study received ethical clearance from the Institutional Ethical Committee of VMMC and Safdarjung Hospital, New Delhi, India. All participants gave written informed consent for their participation in the study. On the day of testing, a detailed medical history was obtained along with demographic and anthropometric variables. Cardiac autonomic function tests included cardiovascular reflex tests and analysis of heart rate variability. Testing was done in the morning after 10 minutes of supine rest in a quiet room with room temperature of 22°C to 24°C. Subjects were instructed to avoid tea or caffeine 12 hours before the test and were asked to report to laboratory at least 2 hours after a light breakfast. Prior to recording, resting blood pressure and heart rate were measured in the comfortable supine position.

#### 2.2.1. Heart Rate Variability (HRV)

HRV parameters were derived from 5-min electrocardiogram (ECG) recordings in lead II configuration in the supine position using Powerlab data acquisition system, AD Instruments. Subjects were asked to relax, breathe normally, and refrain from moving, talking, or sleeping during the procedure. Time domain measures included SDNN and RMSSD, both expressed in milliseconds (ms). Frequency domain HRV indices were calculated using Fast Fourier Transformation and included very low frequency (VLF) power, low frequency (LF) power (0.04-0.15 Hz), high frequency (HF) power (0.15-0.4Hz), and total power (TP). The measurement of TP, VLF, LF, and HF was made in absolute units expressed in ms^2^. However, LF and HF were also measured in normalised units (nu), which represent the relative value of each power component in proportion to the total power minus the VLF component. Normalisation reduces the effect of the changes in total power on the values of LF and HF components and represents balanced behaviour of two divisions of autonomic innervation [11]. The ratio of LF to HF power was also calculated as a measure of sympathovagal balance. SDNN is a measure of combined sympathetic and parasympathetic activity, and RMSSD represents parasympathetic activity. In the frequency domain, LF power indicates a mixture of action of sympathetic and parasympathetic components on heart rate with a predominance of sympathetic ones, whereas HF power reflects parasympathetic modulation of heart rate [12, 13].

#### 2.2.2. Cardiovascular Autonomic Reflex Testing

Cardiovascular reflex tests included deep breathing test and lying to standing test. Heart rate response to deep breathing (delta heart rate and expiration: inspiration ratio) and to standing (30:15 ratio) reflects parasympathetic modulation whereas systolic blood pressure response to standing was used as a measure of sympathetic function. During the tests, ECG in lead II in addition to stethographic respiratory tracings were obtained on student Physiograph, for monitoring of heart rate and respiration, respectively, and automated blood pressure (BP) monitoring was done using Omron device. Brief procedure of reflex tests is as follows [14].

Deep breathing test (DBT): the patient sat quietly and was instructed to breath smoothly, slowly, and deeply at 6 breaths/min. (5 seconds inspiration and 5 seconds expiration), a rate which produces maximum variation of heart rate. Heart rate was measured from ECG. Delta heart rate was the difference between maximal and minimal heart rate during inspiration and expiration, averaged for 6 cycles. E: I ratio was the ratio of longest R-R interval during expiration to the shortest R-R interval during inspiration, averaged over 6 cycles.

Lying to standing test (LST): heart rate and BP responses to standing were recorded. The subject was instructed to stand within 3 s and BP and heart rate were recorded at baseline and at 0.5, 1, 2, and 4 minutes after standing. 30:15 ratio was calculated as the ratio between longest R-R interval at or around the 30th beat and shortest R-R interval at or around the 15th beat. The difference between systolic blood pressure (SBP) at rest and the lowest SBP after standing was recorded.

### 2.3. Statistical Analysis

Statistical analysis was performed by the SPSS program for Windows, version 17.0. Continuous variables are presented as mean ± SD, and categorical variables are presented as absolute numbers and percentage. Data were checked for normality before statistical analysis using Shapiro-Wilk test. Normally distributed continuous variables were compared using ANOVA. If the F value was significant and variance was homogeneous, Tukey multiple comparison test was used to assess the differences between the individual groups; otherwise, Tamhane's T2 test was used. The Kruskal Wallis test was used for those variables that were not normally distributed and further comparisons were done using Mann–Whitney U test. Categorical variables were analyzed using the chi square test. For all statistical tests, a p value less than 0.05 was taken to indicate a significant difference.

## 3. Results

Table 1 shows baseline characteristics of study subjects. The three groups were well matched with regard to age. In addition, preeclamptic group did not differ significantly from the normotensive pregnant women with respect to period of gestation and body mass index. However, women with preeclampsia had significantly higher BMI compared to nonpregnant females. As expected, the values of systolic and diastolic blood pressure as well as heart rate were significantly higher in preeclampsia group compared to normotensive pregnant and nonpregnant females.

**Figure 1:**
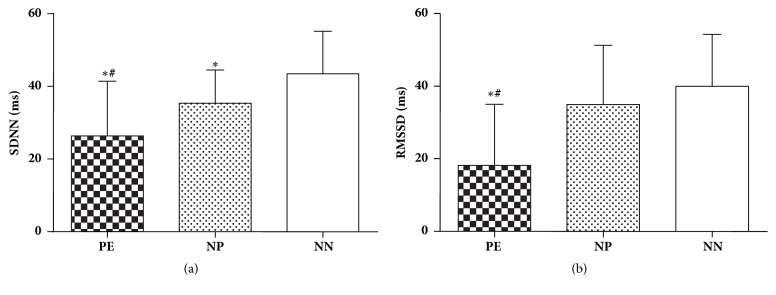
Comparison of (a) SDNN and (b) RMSSD values in 3 groups of subjects; PE, preeclampsia; NP, normotensive pregnant; NN, normal nonpregnant; SDNN, standard deviation of normal R-R intervals; RMSSD, square root of mean squared differences of successive R-R intervals. Values are mean ± SD (n=40). *∗* p < 0.05 versus normal nonpregnant females; ^#^ p < 0.05 versus normotensive pregnant females.

Analysis of time domain parameters of HRV revealed a significantly less SDNN as well as RMSSD in preeclamptic group in comparison to normotensive pregnant and nonpregnant females. Furthermore, value of SDNN in normotensive pregnant females was significantly lower compared to nonpregnant group (Figure 1).

**Figure 2:**
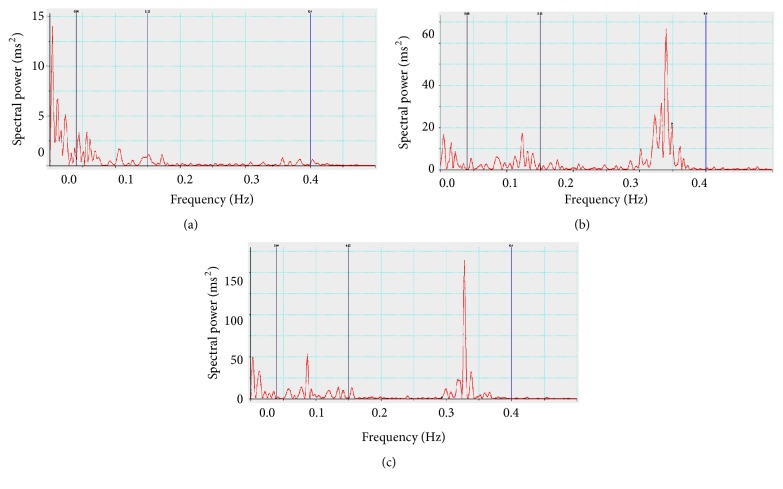
Spectral power of heart rate for different groups. (a) Preeclamptic females; (b) normotensive pregnant females; (c) normal nonpregnant females.

**Figure 3:**
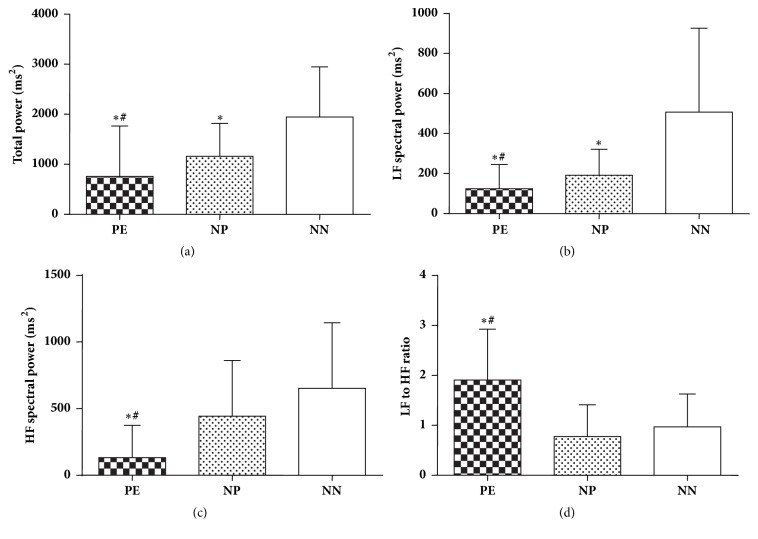
Comparison of spectral components of heart rate variability in 3 groups of subjects; PE, preeclampsia; NP, normotensive pregnant; NN, normal nonpregnant. (a) Total spectral power; (b) LF, low frequency power; (c) HF, high frequency power; (d) LF to HF ratio. Values are mean ± SD (n=40). *∗* p < 0.05 versus normal nonpregnant females; ^#^ p< 0.05 versus normotensive pregnant females.

**Figure 4:**
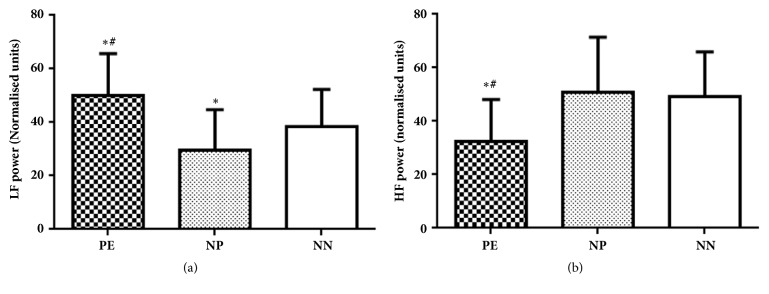
Comparison of spectral components of heart rate variability in normalised units in 3 groups of subjects; PE, preeclampsia; NP, normotensive pregnant; NN, normal nonpregnant. (a) LF, low frequency power and (b) HF, high frequency power. Values are mean ± SD (n=40). *∗* p < 0.05 versus normal nonpregnant females; ^#^ p < 0.05 versus normotensive pregnant females.

Representative recordings of spectrum power of heart rate are depicted in Figure 2. Spectral analysis showed significantly lower values of total power (TP), HF (ms^2^), LF (ms^2^) HF (nu), and higher values of LF nu and LF/HF ratio in preeclampsia group compared to normotensive pregnant as well as nonpregnant group. In addition, normotensive pregnant women exhibited significantly less TP, LF (ms^2^), and LF (nu) compared to nonpregnant women (Figures 3 and 4).

Table 2 depicts the results of cardiovascular reflex tests in different groups. Cardiovagal control as measured by 30: 15 ratio during LST, delta heart rate, and E: I ratio during DBT was significantly reduced in preeclamptic women as compared to normotensive pregnant and nonpregnant groups. However, blood pressure response to standing, which is a measure of adrenergic function, was comparable in all three groups.

## 4. Discussion

Although individual change in autonomic tone and cardiovascular reactivity has been reported in previous studies, to the best of our knowledge, this is the first study in preeclamptic women in which resting cardiac autonomic tone as well as cardiovascular reactivity to stress was evaluated by employing a combination of HRV and cardiovascular reflex tests. The present study demonstrated significant parasympathetic deficit, sympathetic hyperactivity, and sympathovagal imbalance in preeclamptic females compared to normotensive pregnant as well as nonpregnant controls. Parasympathetic decrement is evident from significantly lower values of RMSSD, HF (ms^2^), and HF (nu) in preeclamptic group. Increased LF (nu) points to higher sympathetic influence, whereas higher LF/HF ratio indicates sympathovagal imbalance, contributed by sympathetic dominance and vagal withdrawal in women with preeclampsia. Results of cardiovascular reflex tests demonstrated reduced parasympathetic with a comparable sympathetic control of heart rate in preeclamptic women compared to the other two groups. The three groups were well matched for age and there was no difference of BMI and period of gestation between the two pregnant groups, thus minimising the effects of these confounding factors.

Our findings of HRV are consistent with some of the previous studies on the field [8, 15]. Study by Yang et al. demonstrated lower value of HF and higher LF/HF ratio in preeclamptic women compared to normal pregnant and nonpregnant women [8]. On the other hand, there were studies which had findings in contrast to our results, such as the one by Eneroth and Storck, who observed significantly longer NN intervals but comparable values of frequency domain parameters of HRV in patients with preeclampsia [6]. Furthermore, study by Weber et al. demonstrated increased HRV and higher values of SDNN and RMSSD in women with late but not early onset preeclampsia compared to healthy controls [16]. The authors suggested better autonomic nervous system mediated adaptation in late onset preeclampsia. The discrepant findings of the above two studies could be attributed to relatively small sample size employed in these studies. Besides, Eneroth and Storck evaluated HRV by 24-hour Holter ECG monitoring [6].

Normotensive pregnant females of our study demonstrated significantly lower values of SDNN, TP, LF (nu), and LF (ms^2^) compared to nonpregnant women. Reduction in SDNN and TP signifies a reduction in overall HRV and low LF points to reduced sympathetic modulation of heart rate. These findings are consistent with the results of Stein et al. [17] and Ekholm et al. [18] but not with Greenwood et al. [4] who reported increased central sympathetic output in normal pregnancy. The diverging results could be due to variation in the methodology, as the study by Greenwood et al. [4] measured muscle sympathetic nerve activity by microneurography whereas we have assessed the resting sympathetic tone by analysis of HRV. Regarding cardiovascular reflex tests, our study depicted significantly reduced parasympathetic but no difference in sympathetic modulation of heart rate in preeclamptic women compared to other two groups. Therefore, preeclamptic patients of our study showed reduced vagal tone and an augmented sympathetic drive at rest and a further blunted parasympathetic response to stressful stimuli.

As neural component is a major determinant of peripheral vascular smooth muscle tone, sympathetic hyperactivity, as observed in our study, may be an important contributory factor in development of hypertension and hypoperfusion in preeclampsia. In addition to sympathetic overactivity, reduction in parasympathetic cardiac control also contributes significantly to the pathophysiology of preeclampsia as it has been reported that reproductive age group women have a predominant parasympathetic regulation which plays a crucial role in pregnancy related regulatory resetting, providing protection to healthy pregnancy [7, 19, 20]. With reduced parasympathetic innervation, this protective input is no longer there and, in addition, a heightened sympathetic drive increases vasoconstrictor tone, thus inducing sustained increase in blood pressure. On the basis of the results of the present study we cannot comment conclusively on the possible reason of autonomic derangement in preeclampsia, but we can only speculate on the basis of previous studies. One possible reason may be that, in preeclampsia, intermittent hypoxia associated with dysrhythmic breathing and resultant cardiopulmonary desynchronisation results in sympathetic activation, significantly diminishing vagal balance [19]. Another hypothesis states that, in preeclampsia, the continuous sympathetic centre excitation is a result of enlarged pregnant uterus [20].

Whatever be the reason of autonomic dysfunction, preeclampsia may be associated with serious consequences, increasing both maternal as well as fetal mortality and morbidity [21]. Preeclamptic women may be predisposed to premature cardiovascular disease [22], and the children from preeclamptic pregnancies may have higher risk of stroke, coronary heart disease, and metabolic syndrome in later life [23–25]. Therefore it becomes imperative to effectively manage preeclampsia. Noninvasive assessment of autonomic functions in early part of pregnancy could be of help in prediction, timely diagnosis, and effective management of preeclampsia, thus reducing the risks of side effects and end organ damage in these patients.

The present study has certain limitations that need to be mentioned. Firstly we did not measure serum catecholamines or other methods for assessment of autonomic activity. Secondly, HRV analysis in our study is based on 5-minute ECG recording. Though it is a short length of recording, but it is advocated by Task Force of the European Society of Cardiology and the North American Society of Pacing and Electrophysiology [11]. Besides, HRV and cardiovascular reflex tests used in our study are noninvasive, well established, validated, and risk-free measures of autonomic status. Finally, we did not record respiratory rate during HRV measurements; however, prior to recording subjects were instructed to breathe normally and while recording respiration was monitored by direct inspection.

## 5. Conclusion

The results of our study demonstrate reduction in autonomic vagal modulation and an increase in sympathetic autonomic modulation in preeclampsia. In addition, we observed a reduction of overall HRV in normotensive pregnant women of our study, thereby indicating that even normal pregnancy without any complications is associated with significantly altered impact on autonomic cardiovascular control. Our study strengthens the view that measurement of resting autonomic tone by HRV and reactivity to stressful stimuli, by cardiovascular reflex testing, may be relevant for early screening for preeclampsia as well as for clinical follow-up of patients who are known to have preeclampsia. Cardiovascular reflex tests and HRV are safe, noninvasive measures of autonomic functions and these tests have sufficient sensitivity to detect even subclinical dysautonomia. Reflex tests are time consuming and require considerable patient cooperation. However, HRV analysis is a reliable, easy to use diagnostic tool for autonomic dysfunction.

## Figures and Tables

**Table 1:** Comparison of baseline characteristics in different groups.

**Variable**	**Preeclamptic females (n=40)**	**Normotensive pregnant females (n=40)**	**Normal non pregnant females (n=40)**
Age (years)	26.88 ± 3.52	26.35 ± 2.52	27.52 ± 4.09
Height (cms)	153.4 ± 10.32*∗*	153.6 ± 8.54*∗*	161.2 ± 7.05
Weight (kg)	62.63 ± 8.39	59.42 ± 6.73	62.45 ± 10.58
Body mass index (kg/m^2^)	26.62 ± 2.48*∗*	25.33 ± 3.51	24.01 ± 3.45
Period of gestation (weeks)	30.15 ± 3.55	29.05 ± 2.35	-
SBP (mmHg)	147.38 ± 9.83*∗*^#^	107.2 ± 12.61	110.8 ± 21.00
DBP (mmHg)	93.2 ± 6.76*∗*^#^	84.65 ± 10.92*∗*	76.68 ± 12.22
Heart rate (beats/min)	90.88 ± 14.57*∗*^#^	69.02 ± 12.72	75.82 ± 9.21

Values are expressed as mean ± standard deviation; n, number of subjects; SBP, systolic blood pressure; DBP, diastolic blood pressure; *∗*p < 0.05 versus normal non pregnant females; ^#^*p* < 0.05 versus normotensive pregnant females.

**Table 2:** Cardiovascular reflex tests in different groups.

**Test**	**Parameters**	**Preeclamptic females** **(n=40)**	**Normotensive ** **pregnant females** **(n=40)**	**Normal non pregnant females** **(n=40)**
**Test for sympathetic component**				

**Lying to standing test**	Average fall in systolic blood pressure (mmHg)	3.00 ± 3.96	5.1 ± 6.41*∗*	1.75 ± 2.78

**Tests for parasympathetic component**				

**Lying to standing test**	30:15 ratio	1.13 ± 0.11*∗*^#^	1.22 ± 0.11*∗*	1.40 ± 0.17
**Deep breathing test**	Change in heart rate (bpm)	13.48 ± 6.12*∗*^#^	22.6 ± 8.18	19.82 ± 5.97
	Expiration:Inspiration ratio	1.19 ± 0.10*∗*^#^	1.34 ± 0.12	1.37 ± 0.13

Values are expressed as mean ± standard deviation; n, number of subjects; 30:15 ratio, immediate heart rate response to standing; *∗*p < 0.05 versus normal non pregnant females; ^#^*p* < 0.05 versus normotensive pregnant females.

## Data Availability

The data used to support the findings of this study are available from the corresponding author upon request.
